# Anticancer Properties of Peptide Fragments of Hair Proteins

**DOI:** 10.1371/journal.pone.0098073

**Published:** 2014-06-10

**Authors:** Sergiusz Markowicz, Joanna Matalinska, Katarzyna Kurzepa, Marta Bochynska, Marzena Biernacka, Anna Samluk, Dorota Dudek, Henryk Skurzak, Masaaki Yoshikawa, Andrzej W. Lipkowski

**Affiliations:** 1 Mossakowski Medical Research Centre, Polish Academy of Sciences, Warsaw, Poland; 2 Maria Sklodowska-Curie Memorial Institute and Oncology Centre, Warsaw, Poland; 3 Industrial Chemistry Research Institute, Warsaw, Poland; 4 Research Institute for Production Development, Sakyo-ku, Kyoto, Japan; 5 Tufts University School of Medicine, Boston, Massachusetts, United States of America; University of Tennessee, United States of America

## Abstract

The primary function of hair and fur covering mammalian skin is to provide mechanical and thermal protection for the body. The proteins that constitute hair are extremely resistant to degradation by environmental factors. However, even durable materials can be slowly broken down by mechanical stresses, biodegradation mediated by endogenous enzymes in the skin or host microbes. We hypothesised that the biodegradation products of hair may possess bioprotective properties, which supplement their physical protective properties. Although evolutionary processes have led to a reduction in the amount of hair on the human body, it is possible that the bioprotective properties of hair biodegradation products have persisted. The human skin is exposed to various environmental carcinogenic factors. Therefore, we hypothesised that the potential bioprotective mechanisms of hair degradation products affect melanoma growth. We used pepsin to partially digest hair enzymatically, and this process produced a water-soluble lysate containing a mixture of peptides, including fragments of keratin and keratin-associated proteins. We found out that the mixtures of soluble peptides obtained from human hair inhibited the proliferation of human melanoma cells *in vitro*. Moreover, the hair-derived peptide mixtures also inhibited the proliferation of B lymphoma cells and urinary bladder cancer cells. Normal human cells varied in their susceptibility to the effects of the lysate; the hair-derived peptide mixtures modulated the proliferation of normal human fibroblasts but did not inhibit the proliferation of human mesenchymal cells derived from umbilical cord stromal cells. These results suggest that hair-derived peptides may represent a new class of anti-proliferative factors derived from basically structural proteins. Identification of active regulatory compounds and recognition of the mechanism of their action might pave the way to elaboration of new anticancer drugs.

## Introduction

The mature hair, wool or bristle fiber originates from the cells located at the base of the hair follicle. The genes for two families of structural proteins are activated in matrix, cuticular and cortical cells differentiating in the follicle. These protein families comprise the hair keratins and the hair keratin-associated proteins (KAPs) [1]. KAPs form natural scaffolds that are fulfilled with keratin proteins. The human hair keratin family consists of at least 16 members that are divided into an acidic, type I, and a basic to neutral type II subfamily [2], [3]. Keratin proteins form the 10-nm intermediate filament network of epithelial cells by obligatory association of equimolar amounts of type I and type II keratins [4], [5]. Multiple comparisons of the amino acid sequences derived from the hair keratin genes led to a striking sorting of the keratin proteins into two groups being structurally highly related, and a third containing structurally more heterogeneous hair keratins. Although hair and wool keratins form a very complicated biological composition, their accessibility inspired scientists to consider various applications of hair-derived products in medicine [6]. The enhanced knowledge of physical, chemical and biological properties of keratins over the past years resulted in the elaboration of various new keratin-based biopolymer products, such as films, sponges, scaffolds and fibers [7]. Most of these products starts from substrates produced by oxidative and reductive methods that open disulfide bridges determining three-dimentional structure of proteins [8]. An alternative method recently proposed is the combination of chemical activation and enzymatic digestion [9]. Resulting solid keratin associated proteins scaffolds have been successfully used for various medical and cosmetic applications, including stem cell culture [10]. On the other hand, digestion and solubilization of keratin proteins, and release of peptide fragments may simulate natural mechanism of hair erosion caused by enzymes derived from the skin and/or by enzymes derived from the skin-associated microbes [11]. We hypothesised that the bioprotective peptides produced during hair degradation could have become advantageous during the evolutionary process. Considering the numerous potential functions of hair biodegradation products, we searched for the anticancer effects of these compounds, Our study was focused especially on anti-melanoma effects. Melanoma is the most severe form of skin cancer and accounts for approximately 75 percent of all deaths caused by skin cancers. Exposure to the sun’s UV rays is one of the major factors responsible for somatic mutations that lead to melanoma. Hair and fur cover mammalian skin and provide an important natural physical barrier against UV rays, although in man this physical barrier is less tight than in other mammalians. Skin itself comprise effective neuro- and endocrine systems that effectively protect skin and whole body from harmful environmental elements [12], [13] These systems, including pigment melanin that shields against harmful effects of UV radiation within the human skin, effective in normal conditions, may fail to protect against the excessive exposure to UV rays [14], [15]. We hypothesise, that keratin protein fragments could provide a secondary line of protection against transformed melanoma cells that escaped from the growth control. To test our hypothesis, we obtained a soluble peptide mixture from enzyme-digested human hair [9] and tested the effect of this hair lysate on the proliferation of human melanoma cell lines *in vitro*.

Proteins on the skin may be degradated by endogenous enzymes, serine proteases [16], aspartic proteases [17] and cysteine proteases [18] involved in continual cell replacement in the epidermis as well as enzymes of skin microflora (skin microbiome) [19]. This created great variability of possible combinations of enzymes involved in hair degradation process in homeostatic, acidic condition of the skin. To simulate such combinations we decided to use pepsin, enzyme with quite broad spectrum of selectivity and active in acidic condition.

## Materials and Methods

### Protein Hydrolysates

The study was conducted according to the principles expressed in the Declaration of Helsinki. All participants have been informed on planned experiments and written informed consent was obtained from all donators of hair. All hair specimens were collected after routine haircut and characterized according natural color, participants sex and age. The declaration that hair were not chemically modified has been also provided by participants. The Bioethic Committee of the Warsaw Medical School, Warsaw Poland, approved the experiments, planned procedures, information for participants, and signed participant declarations.

Hair specimens were obtained from two healthy donors, i.e from a partially grey blond elderly male (OM) and from a young brunette woman (YW). Hair specimens of each donor were separately processed to obtain hair lysate. Hair were chemically pre-activated with sodium hydroxide and digested with pepsin following general procedure published previously [9]. Donated hair were chopped to small fragments and suspended in 3% solution of NaOH for 1 hour at room temperature (21°C). Next, the hair was filtered off and washed twice with water. Then, hair was suspended in water and acidified with 1% hydrochloric acid to pH 1.6, followed by addition of pepsin of activity jFiP 1200/g of 0.3% by weight of the starting amount of hair. The mixture was heated to 40°C and the acidity of the reaction mixture was controlled to maintain pH 1.6–2.1 range by the appropriate addition of 1 N hydrochloric acid. After 12 hours, the remaining precipitate was filtered off and washed with water. The collected filtrates were heated to 80°C and rapidly cooled to room temperature (21°C), than extracted with ethyl ether, frozen and freeze-dried. Unsolubilized residual material was dried and grounded to small fragments (<0.5 mm) and digested again with pepsin at pH 1.6 for 12 hours at 40°C. Filtrates were isolated and elaborated as described previously. Dry materials from first and second hydrolyses were collected and solubilized in 70% ethanol. Small amount of unsoluble residue was filtered out. Filtrate has been evaporated to dryness and used in biological tests. Yield 14% of starting amount of hair. Calculated mean size of peptides 3.5, calculated on the basis of total % N 8.72 and free amino groups % N 2.11.

### Cell Cultures

The established human melanoma cell lines MeW100, MeW151, MeW152, MeW155, MeW156, MeW164, MeW168, MeW175, MeW184, MeW186, and MeW187 were obtained from the institutional repository at the Maria Sklodowska-Curie Memorial Institute and Oncology Centre in Warsaw [20]–[23]. FTSLC melanoma cell line was a gift from Dr Monika Vetterlein from the Univ. of Vienna [24]. Human urinary bladder carcinoma cell line T24 was a gift from prof. Georg Klein from the Karolinska Institute in Stockholm [25]. Human fetal fibroblast cell line FlWp95 and normal adult human fibroblast cell lines Fib9 and FlW180 were obtained from the institutional repository at the Maria Sklodowska-Curie Memorial Institute and Oncology Centre in Warsaw [21], [23]. The melanoma cell lines, the urinary bladder cancer cell line and the fibroblast cell lines were cultured in Eagle’s 1959 MEM (Biomed-Lublin) supplemented with 10% foetal calf serum (Invitrogen), 50 µg/ml penicillin G, 50 µg/ml streptomycin, and 0,1% glutamine in a humidified atmosphere with 5% CO_2_.

Umbilical cord stromal cells (UCSC) isolated from Wharton’s jelly of umbilical cord were a gift from prof. Zygmunt Pojda [26] from the Stem Cell and Tissue Bank in the Maria Sklodowska-Curie Memorial Institute and Oncology Centre in Warsaw. The Wharton’s jelly was separated from the cords obtained from physiological full time deliveries. Isolated mesenchymal cells (UCSC) were cultured and passaged in CellGro SCGM medium for serum-free culture of hematopoietic stem and progenitor cells (CellGenix).

Follicular lymphoma cell line DOHH-2 was purchased from the German Collection of Microorganisms and Cell Cultures (DSMZ), Braunschweig, Germany). Burkitt lymphoma cell line Namalwa was a gift from prof. Georg Klein form the Karolinska Institute in Stockholm [27], [28]. Normal human peripheral blood lymphocytes lymphocytes were isolated from buffy coats purchased from the Regional Blood Center in Warsaw. Human B cell lymphoma lines DOHH-2 and Namalwa as well as normal human lymphocytes isolated from the peripheral blood were cultured in AIM V medium (Invitrogen).

### Generation of Melanoma Cell Sublines Derived from Gamma Irradiation-resistant Melanoma Cells

Melanoma cell lines FTSLC, MeW100, MeW152 were exposed to a single high dose of radiation from a ^137^Cs source (30 Gy, 60 Gy, 90 Gy or 120 Gy) and plated in culture flasks for 4–5 days. Adherent cells were harvested with the use of 0.25% trypsin with EDTA (200 mg/L) (Sigma) and passaged. Melanoma sublines were established from a minor cell subpopulations proliferating following the passage of irradiated melanoma cells. MeW100 and MeW152 sublines were generated from the cells resistant to 30 Gy, and FTSLC subline from the cells resistant to 60 Gy of gamma irradiation.

### Cell Proliferation Assays

Adherent cell cultures were plated in 24-well plates. The initial cell number in culture and timing of medium supplementation was adjusted for each cell line to achieve the optimal cell growth. The culture timing was adjusted to harvest cells at the state of sub-confluency. Adherent cells were harvested using 0.25% trypsin with EDTA (200 mg/L). Following trypsinization, the harvested cells were counted directly in a haemacytometer under a microscope, and cell viability was evaluated according to trypan blue staining. Lymphoma cells and normal lymphocytes cultured in suspension were harvested at the logarithmic phase of growth. The harvested cells were counted directly in a haemacytometer under a microscope. Cell viability was evaluated according to trypan blue staining.

In the selected experiments, cell proliferation was also measured by flow cytometry in the CFSE dilution assay using the CellTrace CFSE Cell Proliferation Kit (Invitrogen) to stain the cells. Prior to setting of the cultures with or without lysates, cells were suspended in PBS Ca^++^Mg^++^ free/0.1% HSA with 1 µM carboxyfluorescein diacetate, succinimidyl ester (CFSE) and incubated for 15 min at 37°C. The staining was quenched by addition of ten volumes of culture medium and 30′ incubation in room temperature in dark. Cells were pelleted by centrifugation, washed twice and suspended in the respective culture medium. The proliferation of CFSE-stained cells collected after the culture was evaluated by both direct cell counting under a microscope and by flow cytometry using the FACSCalibur (Becton-Dickinson).

## Results

**Figure 1:**
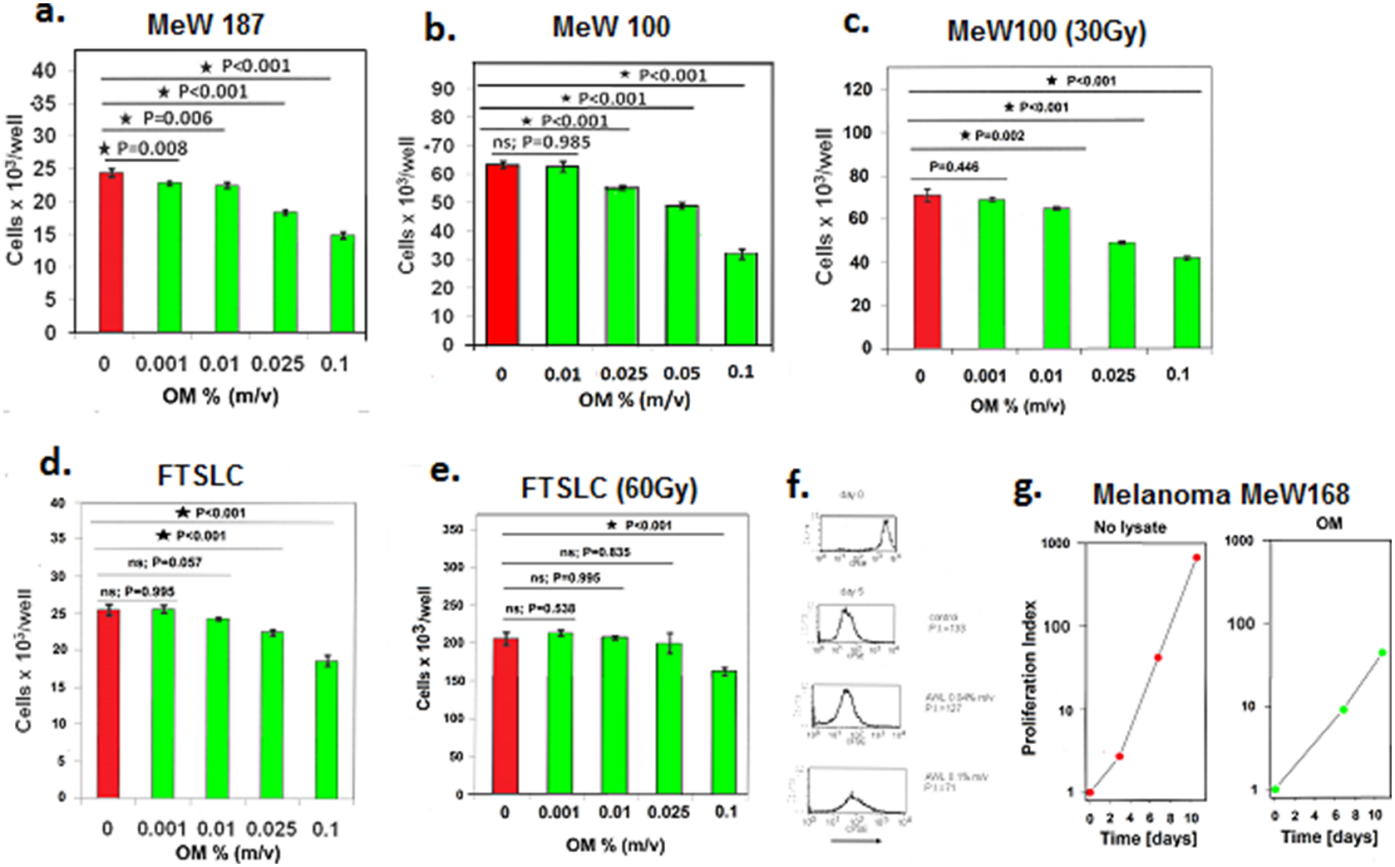
The human hair lysate obtained from the OM male donor inhibited the proliferation of early and late-passage human melanoma cells. Gamma irradiation-resistant melanoma sublines generated from these cells remained susceptible to the inhibitory effects of the hair lysate. The viable cell count was evaluated in 3-day cultures of MeW187 melanoma cells plated at 13×10^3^ cells/well with or without the lysate following 3 previous passages (a); 4-day cultures of the MeW100 melanoma cells plated at 7×10^3^ cells/well following 38 previous passages (b); 4-day cultures of the Mew100 subline pre-selected by irradiation with a ^137^Cs source (30 Gy) and plated at 10×10^3^ cells/well (c); 3-day cultures of FSTLC melanoma cells plated at 15×10^3^ cells/well after 53 passages (d); and 3-day cultures of the FSTLC subline preselected by irradiation (60 Gy) and plated at 15×10^3^ cells/well (e). Fresh medium with or without the lysate was added to the cultures once daily. The groups of cultures treated with the lysate were compared to the control cultures using Dunnett’s test. Arrows mark the initial cell number in the cultures, and the bars represent the mean values ± s.d. of the viable cell count in triplicate cultures. Flow cytometry analysis of CFSE-labelled cells demonstrated that there were fewer cell divisions in the melanoma cell cultures supplemented with the lysate than in the control group (f). The histograms of the CFSE dilutions in MeW155 melanoma cells cultured for 5 days with or without the lysate are representative of triplicate cultures. The lysate was added only once at the start of the 5-day culture following labelling with CFSE. The proliferation indices (P.I.) were calculated as the proportion of the mean final cell yield to the initial cell number in the cultures. The effects of the lysate were evaluated in the prolonged passaged cultures (g). Proliferation indices were determined for MeW168 melanoma cells cultured with or without OM lysate on days 3 and 7 when the cells were passaged, and on day 11. Fresh medium containing the lysate at a concentration of 0.1% (m/v) was added on days 0, 3, 7, and 9.

The human hair lysate inhibited the proliferation of early and late-passage human melanoma cell lines (examples in Fig. 1a, b, d). To determine whether the hair lysate was also able to regulate gamma irradiation-resistant melanoma cell lines, we generated melanoma sublines derived from minor subpopulations of melanoma lines that were able to proliferate following the exposure to a single high dose of radiation from a ^137^Cs source. Three melanoma sublines were generated, and two of these sublines were inhibited by the hair lysate to the same extent as the original melanoma lines (example in Fig. 1b, c). The antiproliferative effect of the lysate on the subline derived from irradiation-resistant FLTCS melanoma cells was significant but less pronounced than the effect on the original FLTCS line (Fig. 1d, e). As a result of the selection by irradiation, the FLTSC subline acquired a much higher proliferative potential, as well as a distinct cellular morphology, than the original FLTSC line, and therefore the measurement of the lysate effect on FTSLC subline could be biased. At the given lysate concentration used in the culture medium, the rapidly expanding cells of the irradiation-selected FLTSC subline were exposed to a lower proportion of the lysate during the culture than the slowly proliferating cells of the original FLTSC line.

Flow cytometry analysis of carboxyfluorescein succinimidyl ester (CFSE)-labelled cells revealed that the number of cell divisions was reduced in hair lysate-cultured melanoma cells as compared to the control cultures (example in Fig. 1f). In addition, when the lysate was repeatedly added to the culture medium and the cells were passaged to avoid cell confluency during the prolonged culture period, the yield of the melanoma cells cultured in the presence of the lysate was continuously lower than the yield from control cells cultured without the lysate (Fig. 1g). However, the regulatory effect of the lysate on melanoma cell proliferation was reversible if the hair lysate was removed from the culture medium (data not shown).

**Figure 2:**
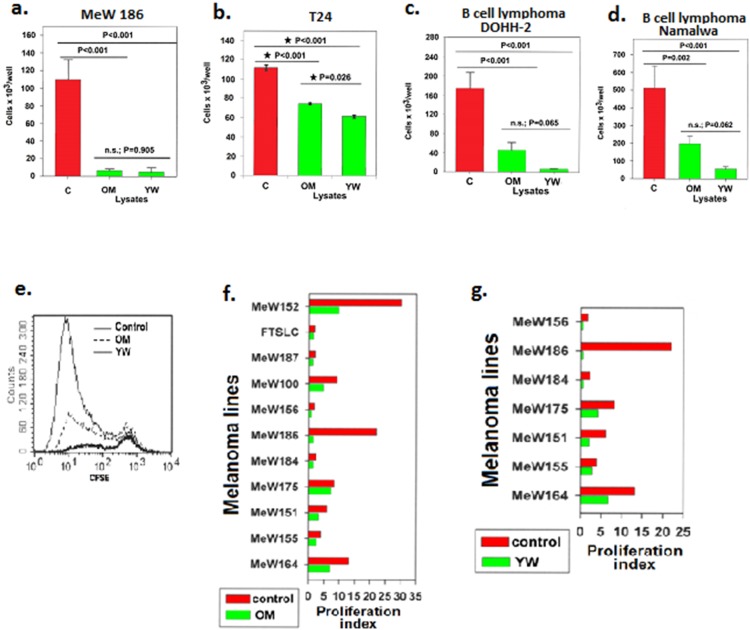
The OM and YW human hair lysates obtained from two different donors inhibited the proliferation of human melanoma cells and other types of human cancer cells *in vitro*. The viable cell count was evaluated in 7-day cultures of MeW186 melanoma cells plated after 3 passages at 5×10^3^ cells/well with or without the hair lysates (a); 4-day cultures of urinary bladder cancer T24 cells plated at 5×10^3^ cells/well (b); 4-day cultures of the B cell lymphoma line Namalwa plated at 120×10^3^ cells/well (c); and 4-day cultures of the B cell lymphoma line DOHH-2 plated at 120×10^3^ cells/well (d). The lysates were added at a concentration of 0.1% (m/v). Fresh medium with or without the lysate was added to the MeW186 melanoma cell cultures on days 0, 3, 4, and 5. Medium with or without the lysate was changed once daily for cultures of the urinary bladder cancer T24 cells and for cultures of lymphoma cells. The bars represent the mean values ± s.d. of the viable cell count in triplicate cultures. Groups of cultures were compared using the LSD ANOVA test. The profiles of the CFSE dilution in DOHH-2 cells indicated that the lymphoma cells divided in the presence of the lysate, although the yield of viable cells was substantially reduced in comparison to cells cultured in the absence of the lysate (d); the representative histograms for cultures shown in Fig. 2d. A comparison of the proliferation indices showing that both the OM lysate (f) and the YW lysate (g) inhibited the proliferation of various melanoma lines generated from different patients. The proliferation index was calculated as the proportion of the mean viable cell count following culture to the viable cell number at the beginning of the culture.

Furthermore, two different hair lysates, one obtained from an old man with partially grey blond hair (OM) and the other obtained from a young brunette woman (YW), effectively inhibited the proliferation of human melanoma cells (Fig. 2a), urinary bladder cells (Fig. 2b), and B lymphoma cells (Fig. 2c, d) when used at a concentration as low as 0.1% m/v. The B lymphoma cells maintained the capacity to divide in the presence of the hair lysates, but the rate of cell division was reduced in comparison to that of the control cultures, as determined by flow cytometry analysis of CFSE-labelled cells (a representative experiment is shown in Fig. 2e).

Next, a comparison of the proliferation rate of cells cultured with and without the hair lysate at a concentration of 0.1% m/v demonstrated that the OM lysate inhibited the proliferation of 11 melanoma lines obtained from different patients (Wilcoxon Signed-Rank Test analysis; P = 0.003) (Fig. 2f) and that the YW lysate inhibited the proliferation of 7 melanoma cell lines (Wilcoxon Signed-Rank Test analysis; P = 0.018) (Fig. 2g).

**Figure 3:**
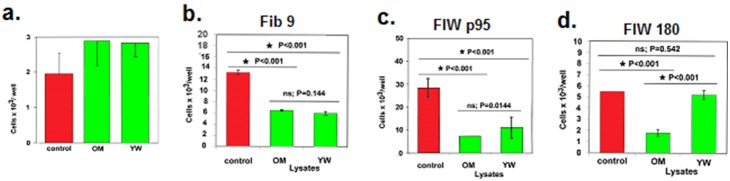
The effect of human hair lysates on the proliferation of normal human cells. The OM and YW human hair lysates, when used at a concentration of 0.1% (m/v), did not significantly affect the proliferation of normal human mesenchymal cells derived from the umbilical cord (a). The antiproliferative effect of the lysates on fibroblast lines was dependent on the lysate tested and on the fibroblast line tested (b, c, d). Groups of cultures plated in triplicate were compared using the LSD ANOVA test.

Normal human cell populations varied in their susceptibility to regulation by the human hair lysates. For example, the human hair lysates did not significantly affect the proliferation of mesenchymal cell line obtained from the umbilical cord (Fig. 3a). In addition, depending on the human fibroblast line tested, the OM lysate and the YW lysate equally inhibited the proliferation of the human fibroblast lines Fib 9 and FlWp95 (Fig. 3b, c), and only the OM lysate was able to regulate the proliferation of the fibroblast line FlW 180 (Fig. 3d). Moreover, the OM lysate partially inhibited the proliferation of T cells that were activated polyclonally with anti-CD3 monoclonal antibodies, whereas the YW lysate stimulated the proliferation of these T cells (data not shown).

Lysates produced from fur of other mammalian species differently affect human melanoma cell proliferation (results not shown), which suggests that the activity of hair lysates is species-specific.

## Discussion

Numerous studies have suggested that membrane-active peptides involved in the innate immune defence against microbial pathogens can extend their cytotoxic activity to cancer cells [29]–[33]. Endogenous mammalian anti-microbial host defence peptides, such as defensins and peptides generated from bovine lactoferricin through pepsin cleavage or various anti-microbial peptides derived from evolutionary distant organisms, have been shown to have activity against mammalian cancer cells. Because of the high content of negatively charged components in the cancer cell membrane, cancer cells are more susceptible to interactions with cationic amphiphilic peptides, which lead to perturbation of the cell membrane bilayer. The activity of such membrane-active peptides leads to cell lysis, necrosis and/or apoptosis. Endogenous peptides can also act through defined signalling molecules, as the endogenous peptide hormone angiotensin (1–7) indirectly inhibits cancer growth by targeting the receptors and factors involved in the regulation of angiogenesis [34]. Alternatively, peptides may induce the response of the immune system to create a hostile environment for cancer cells in the body [35], [36]. In general, anticancer peptides have various cell function blocking properties, including interactions with cell membrane receptors, modulation of cell adhesion through interactions with extracellular matrix proteins, inhibition of protein kinases and proteases as well as antiangiogenic activity [37].

Our study provided evidence that human hair protein lysates possess antiproliferative properties that may be involved in the natural protection against cancer. These lysates are composed of a mixture of various peptides, including fragments of hair structural proteins Therefore, the combination of the individual activities of the peptides present in the lysate represents the total observed activity of the lysate. In addition, it is likely that these lysates contain a spectrum of various peptide activities, including opposite activities. Human hair degradation products regulate cancer cell proliferation, but can also regulate proliferation of fibroblasts. It suggests that hair degradation products may affect the expansion of cancer-associated fibroblasts involved in the cancer promotion in the cancer microenvironment, but also the proliferation of normal fibroblasts involved in the process of normal tissue regeneration. However, the anti-proliferative activity of the hair-lysate does not extend on the mesenchymal cell population derived from UCSC. The question is to be answered if hair-derived peptides could inhibit over-proliferation of fibroblasts leading to tissue fibrosis.

Regarding the abundance of keratins and keratin-associated proteins in the skin, it is possible that products of degradation of these proteins significantly regulate cancer growth in the cancer microenvironment. At the lysate concentrations used in the current study, the proliferation of cancer cells was inhibited *in vitro*, although viable cancer cells were able to persist in the cultures. Therefore, it is possible that hair protein fragments may play a role in keeping tumour growth at bay if used at a concentration that does not kill cancer cells, but regulates their proliferation. The antiproliferative effects of the hair lysates were observed only when the lysates were used at relatively high concentrations in the culture medium. A proportion of active anti-cancer components in the lysate is unknown yet. Thus, it will be interesting to determine whether purified active anti-cancer components of the lysate are directly cytotoxic to cancer cells when used at higher concentrations, and whether the regulation of cancer growth by the degradation products of hair structural proteins differs from that mediated by highly cytotoxic peptides which primarily have a defensive function.

It was reported that peptide defensins released by granulocytes can contribute to extracellular cytotoxicity against various tumour targets but are markedly inhibited by albumin and other macromolecular serum components [38], [39]. Therefore, it should be taken into account that we studied the antiproliferative effects of hair lysates on cells that were cultured either with foetal calf serum or with human albumin, depending on the respective cell requirements. Thus, we cannot exclude the possibility that serum proteins used in the culture medium could have partially neutralised the effects of the lysates, although the lysate activity was clearly evident.

Our study sought to identify orally active peptides that have marked resistance to digestive degradation. Therefore, the present work was focused on pepsin lysates. Additionally, it remains unclear whether particular anticancer peptides can be isolated from the lysates and whether these will retain their activity after oral consumption. Further studies on the isolation and characterisation of individual peptides are in progress.

## Conclusions

We attempted to verify the hypothesis that the biodegradation products of human hair may be involved in the natural protection against cancer in the in skin exposed to carcinogenic environmental factors. A water-soluble mixture of peptides obtained in the process of partial enzymatic digestion of human hair inhibits proliferation of human melanoma cells *in vitro*. Such peptide mixtures inhibits also the proliferation of B lymphoma cells and urinary bladder cancer cells. These results indicate that hair-degradation products contain a new class of active anti-proliferative compounds that may regulate cancer growth. The hair-derived peptides modulate proliferation of normal fibroblasts, but do not inhibit proliferation of normal mesenchymal cells from umbilical cord stromal cells. Hair-degradation products containing fragments of keratin and keratin-associated proteins are a potential source of new anti-cancer drugs. Attempts to isolate active anti-proliferative compounds and to delineate their selectiveness toward cancer versus normal cell populations are undertaken.
